# Multiple plasmid origin‐of‐transfer regions might aid the spread of antimicrobial resistance to human pathogens

**DOI:** 10.1002/mbo3.1129

**Published:** 2020-10-27

**Authors:** Jan Zrimec

**Affiliations:** ^1^ Department of Biology and Biological Engineering Chalmers University of Technology Gothenburg Sweden

**Keywords:** antimicrobial resistance, bioinformatics, DNA structure prediction, horizontal gene transfer, microbial ecology, plasmid conjugation

## Abstract

Antimicrobial resistance poses a great danger to humanity, in part due to the widespread horizontal gene transfer of plasmids via conjugation. Modeling of plasmid transfer is essential to uncovering the fundamentals of resistance transfer and for the development of predictive measures to limit the spread of resistance. However, a major limitation in the current understanding of plasmids is the incomplete characterization of the conjugative DNA transfer mechanisms, which conceals the actual potential for plasmid transfer in nature. Here, we consider that the plasmid‐borne origin‐of‐transfer substrates encode specific DNA structural properties that can facilitate finding these regions in large datasets and develop a DNA structure‐based alignment procedure for typing the transfer substrates that outperforms sequence‐based approaches. Thousands of putative DNA transfer substrates are identified, showing that plasmid mobility can be twofold higher and span almost twofold more host species than is currently known. Over half of all putative mobile plasmids contain the means for mobilization by conjugation systems belonging to different mobility groups, which can hypothetically link previously confined host ranges across ecological habitats into a robust plasmid transfer network. This hypothetical network is found to facilitate the transfer of antimicrobial resistance from environmental genetic reservoirs to human pathogens, which might be an important driver of the observed rapid resistance development in humans and thus an important point of focus for future prevention measures.

## Graphical abstract

A structural alignment procedure developed here enables finding and typing plasmid‐borne origin‐of‐transfer substrates in large plasmid datasets. Thousands of putative DNA transfer substrates are identified, showing that plasmid mobility can be twofold higher and span almost twofold more host species than is currently known. Over half of all the putative mobile plasmids carry conjugation systems belonging to different mobility groups. This plasmid transfer network might facilitate the transfer of antimicrobial resistance from environmental genetic reservoirs to human pathogens.

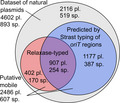

## 1 INTRODUCTION

Horizontal gene transfer of antimicrobial resistance (AMR) genes occurs via the processes of transformation and conjugation. The former mediates especially narrow range, intra‐genus transfers (Gibson et al., 2015; Hu et al., 2016), whereas the latter is implicated in a wider range of transfer hosts (Garcillán‐Barcia et al., 2011; Zrimec & Lapanje, 2018) and potentially enables AMR to overcome the toughest phylogenetic and ecological transmission barriers (Ben Maamar et al., 2020; Dolejska & Papagiannitsis, 2018; Malhotra‐Kumar et al., 2016; Mathers et al., 2015; Sun et al., 2019; Wang & Sun, 2015). Consequently, the interaction between conjugative relaxase enzymes and their DNA origin‐of‐transfer (*oriT*) substrates facilitates the majority of all AMR transfers in nature (Alekshun and Levy, (2007); Wintersdorff et al., 2016) and is especially important for ones related to human infection complications (San Millan, 2018). However, the current knowledge on conjugative transfer mechanisms and systems (Fernandez‐Lopez et al., 2017; Garcillán‐Barcia et al., 2009; Smillie et al., 2010a; Zrimec & Lapanje, 2018) is unable to describe the unprecedented amount of observed horizontal transfer (Lopatkin et al., 2017; Mathers et al., 2015; Sun et al., 2019; Wintersdorff et al., 2016) that seems to transcend all transfer barriers between resistance reservoirs and human hosts (Ben Maamar et al., 2020; Dolejska & Papagiannitsis, 2018; Malhotra‐Kumar et al., 2016; Salyers & Amábile‐Cuevas, 1997; San Millan, 2018; Sun et al., 2019; Wang & Sun, 2015; Wintersdorff et al., 2016).

The standard approach for characterization of plasmid mobility involves the classification of conjugation and mobilization genes (Smillie, et al., 2010), especially typing of relaxase enzymes into the respective mobility (Mob) groups (Garcillán‐Barcia et al., 2009; Garcillán‐Barcia et al., 2020). However, besides the possibility of yet unidentified enzymes and mobility groups (Coluzzi et al., 2017; Garcillán‐Barcia et al., 2009; Guzmán‐Herrador & Llosa, 2019; Ramachandran et al., 2017; Soler et al., 2019; Wisniewski et al., 2016), multiple new processes have recently been uncovered that might confer additional mobility to plasmids and involve the *oriT* substrate. These include (aa) broadened relaxase‐binding specificities to multiple different *oriT* sequence variants (Chen et al., 2007; Fernández‐González et al., 2016; Fernández‐López et al., 2013; Jandle & Meyer, 2006; Kishida et al., 2017), which, according to the evolutionary theory of such DNA regions (Becker & Meyer, 2003; Parker et al., 2005; Zrimec & Lapanje, 2018), indicates the possibility of plasmids carrying multiple functional secondary *oriT*s, and (b) trans‐mobilization of plasmids carrying *oriT*s triggered by relaxases from co‐resident plasmids acting *in trans* on the non‐cognate *oriT*s (Moran & Hall, 2019; O’Brien et al., 2015; Pollet et al., 2016; Ramsay & Firth, 2017). The latter mechanism demonstrates that *oriT* regions are the only elements of the conjugation machinery required in *cis* (Guzmán‐Herrador & Llosa, 2019) and suggests that many plasmids classified as non‐mobile due to the absence of putative relaxases may be mobilizable (Ramsay & Firth, 2017). However, although typing the *oriT* enzymatic substrates instead of the genetic scaffolds might present improvements to the current understanding of plasmid mobility, no systematic studies of *oriT*s across sequenced plasmids have yet been performed, likely due to the lack of available data and tools that would enable such *oriT* typing.

A major problem with uncovering *oriT* regions is that, apart from being experimentally laborious, it is computationally challenging due to multiple molecular mechanisms and a variety of DNA sequence elements present and coevolving in the DNA substrate (Zrimec & Lapanje, 2018), even among plasmids belonging to a single species such as *Staphylococcus aureus* (O’Brien et al., 2015). The *oriT* region contains recognition and binding sites for the relaxase enzyme as well as accessory proteins that help to initiate mobilization. These sites include inverted repeats and hairpins (Frost et al., 1994; Sut et al., 2009; Williams & Schildbach, 2007) as well as the nicking site *nic*, where relaxase cleaves the DNA to initiate plasmid transfer (Frost et al., 1994). They are characterized by specific DNA physicochemical and conformational features that underpin key protein‐DNA readout and activity mechanisms (Kolomeisky, 2011; Rohs et al., 2009, 2010; Zrimec & Lapanje, 2015, 2018) as well as define conserved niches of structural variants that enable good resolution between Mob groups and subgroups (Zrimec & Lapanje, 2018). *OriT* typing thus requires algorithms beyond simple sequence‐based alignment (Altschul et al., 1990; Li et al., 2018) that can recognize and process the more complex molecular motifs and underlying DNA physicochemical and conformational (i.e., structural) features. The use of DNA structural representations has indeed led to improvements in algorithms for the identification of other regulatory regions, such as promoters and replication origins (Abeel et al., 2008; Bansal et al., 2014; Chen et al., 2012; Dao et al., 2018; Samee et al., 2019). Despite this, instead of using tools that probe the actual relaxase‐*oriT* interaction potential by identifying molecular properties that are the basis of such interactions, conventional approaches for *oriT* analysis still rely on sequence alignment‐based methods (Altschul et al., 1990; Li et al., 2018; O’Brien et al., 2015).

Here, we prototype a DNA structure‐based alignment algorithm for finding *oriT* variants, which enables finding and also Mob‐typing *oriT* regions across thousands of sequenced plasmids. Based on the newly uncovered *oriT* variants, since they can facilitate both *in cis* and *in trans* plasmid transfer, the amount of putative mobile plasmids and putative mobile plasmid‐carrying host species is re‐analyzed. We then evaluate if and how the uncovered fraction of *oriT*s might help to overcome the known barriers to horizontal gene transfer, by reconstructing and analyzing a hypothetical network of potential AMR transfers between different species and habitats, especially those from the environmental reservoir to the human microbiota.

## 2 METHODS

### 2.1 M1. Datasets used for alignments

**FIGURE 1:**
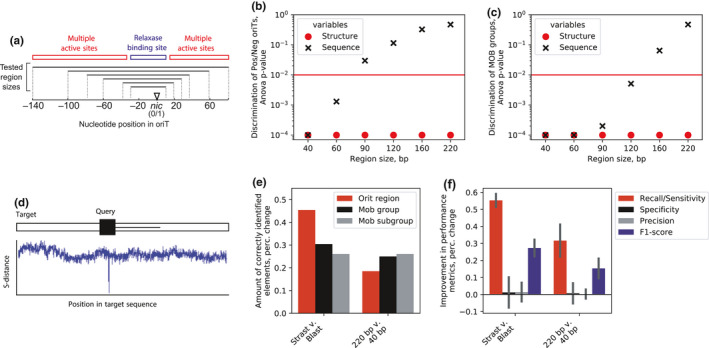
The structural alignment algorithm improves *oriT* typing performance. (a) Schematic depiction of the *oriT* region and different analyzed *oriT* subsets of lengths 40, 60, 90, 120, 160, and 220 bp, which span the single relaxase‐binding site or multiple protein recognition and binding (ie. ‘active’) sites, respectively. (b, c) Statistical analysis of nucleotide and structural representations (Methods M3) with different *oriT* subsets for (B) Mob group discrimination and (c) discrimination of positive and negative examples. (d) Schematic depiction of the structural alignment algorithm, which finds the positions in the target dataset with minimum s‐distance to the query sequences. (e) Comparison of the amount of correctly identified elements between our algorithm (Strast) and Blast, and by using 220 or 40 bp *oriT* subsets, for *oriT* typing as well as discrimination of MOB groups and subgroups. Error bars denote 95% confidence intervals. (f) Comparison of machine learning performance metrics between our algorithm (Strast) and Blast, and by using 220 or 40 bp *oriT* subsets. Error bars denote 95% confidence intervals.

The full query dataset with known *nic* sites comprised 112 distinct *oriT* regions from 118 plasmids, where a single *oriT* sequence was selected to represent *oriT*s with sequence similarity below 15%, and 6 Mob groups {F,P,Q,V,C,T} (Table A1‐1, Dataset S1: https://github.com/JanZrimec/oriT-Strast/blob/master/data/Dataset_S1.csv). The dataset included (i) 48 experimentally verified *oriT* regions, of which 34 contained experimentally verified nicking sites and 14 contained putative nicking sites, and (ii) 59 *oriT* regions with computationally predicted nicking sites. The part of the *oriT* with relevant protein binding features from −140 to +80 bp according to the *nic* site was used (Figure 1a).

For initial development and testing of the structural alignment algorithm, due to the lack of a sufficient number of elements from Mob groups C, H, and T for correct testing (below 10 elements per group), a 4 Mob group {F,P,Q,V} version of the query dataset with 106 elements was used (Table A1‐1). The balanced dataset from 4 Mob groups {F,P,Q,V} used for s‐distance testing was a subset of the query dataset containing approx. 16 elements from each Mob group (Zrimec & Lapanje, 2018; Table A1‐1). A set of negative examples was obtained for each element by extracting sequences from the neighboring vicinity of *oriT*s. Specifically, the negative examples were selected randomly from a region 200 to 800 bp upstream and downstream from experimental *nic* sites, thus containing different non‐*oriT* coding and non‐coding regions with low sequence similarity (p‐distance >0.6). The testing datasets included (a) 51 plasmids with known *oriT* locations and Mob groups but unknown *nic* sites (Dataset A2: https://github.com/JanZrimec/oriT-Strast/blob/master/data/Dataset_S2.csv) and (b) 13 plasmids with 14 experimentally determined *nic*/*oriT* sites but unknown Mob groups, obtained from the OriTDB database (Li et al., 2018; Table A1‐3).

### 2.2 M2. Development and testing of alignment algorithms

**FIGURE A1:**
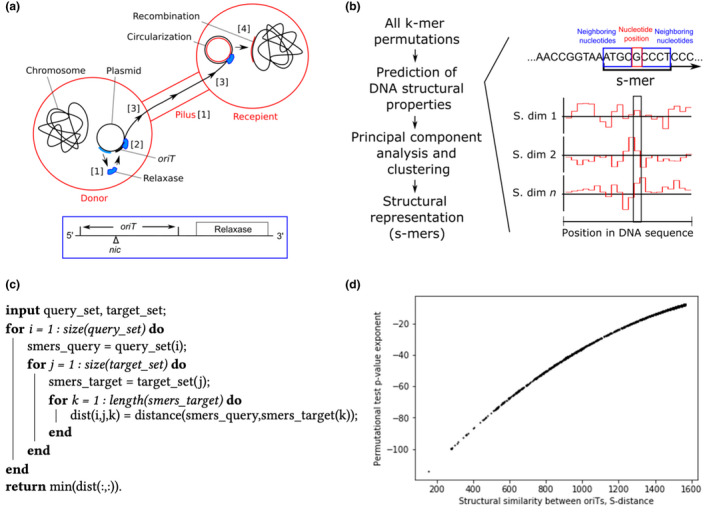
Overview of the structural alignment framework. (a) Depiction of the plasmid conjugation process, which can be divided into 4 steps: (i) formation of a conjugative pilus that connects the donor and recipient cells for transmission of mobile DNA, (ii) expression of enzymes (e.g., relaxase) and accessory proteins, which recognize the binding sites at the DNA origin of transfer (*oriT*), where plasmid transfer is initiated, (iii) relaxase cuts into the *oriT* at the *nic* site, exposes the single‐stranded DNA and, with the help of the protein transport system, transfers DNA to the recipient cell, (iv) in the recipient, either the missing DNA strand is synthesized and then circularized, in case of plasmid transfer, or the mobile DNA is integrated into the chromosome by recombinant mechanisms, whereas in the donor cell reconstruction of the missing DNA occurs. (b) Depiction of the encoding of DNA into structural representations, where consecutive k‐mers of the DNA (of length 7 bp) are encoded with clustered DNA structural property embeddings (marked s. dim. 1, s. dim. 2, …, s. dim. *n*; *n* = 18 such embeddings used) into a compressed structural representation termed 's‐mers'. To compute the s‐mers, 64 DNA structural properties were predicted for all permutations of nucleotide k‐mers, after which principal component analysis (first 18 components with >99% of data variance were used) and clustering (number of clusters 128) were performed. (c) Pseudocode giving an outline of the sequence alignment framework, which allows the use of the s‐distance measures between the target and query sequences. The s‐distance is the Euclidean distance between all respective embeddings of two such structurally encoded DNA sequences. The algorithm takes as input a set of query and target sequences, and for each query and target sequence, encodes them into structural representations, and returns the regions in the target sequence with the lowest s‐distance to the query sequence. (d) Mapping of s‐distance scores to *p*‐values obtained using permutational (bootstrap) tests, where bootstraps of the query *oriT* sequences were used to estimate *p*‐values at cutoffs from 1e‐6 to 1e‐1. These points together with the theoretically predicted limit ~1e‐132 were then used to fit to a second‐order polynomial function (f = p_0_ᐧ x^2^ + p_1_ᐧx + p_2_; p_0_ = −3.045e‐05, p_1_ = 0.128, p_2_ = −133.000).

A DNA structure‐based alignment algorithm, termed Strast, was developed and tested. The algorithm: (a) takes as input a set of query and target DNA sequences, (b) encodes the input query and target DNA sequences into structural representations (Figure A1‐1b), and (c) finds and returns the most similar segments of target sequences to query sequences based on a structural distance measure (s‐distance, Figure A1‐1c: algorithm pseudocode). The practical implementation of the algorithm uses precomputed parameters for structurally encoding the DNA sequences as well as a precomputed distance matrix for computing the s‐distance function.

**FIGURE A2:**
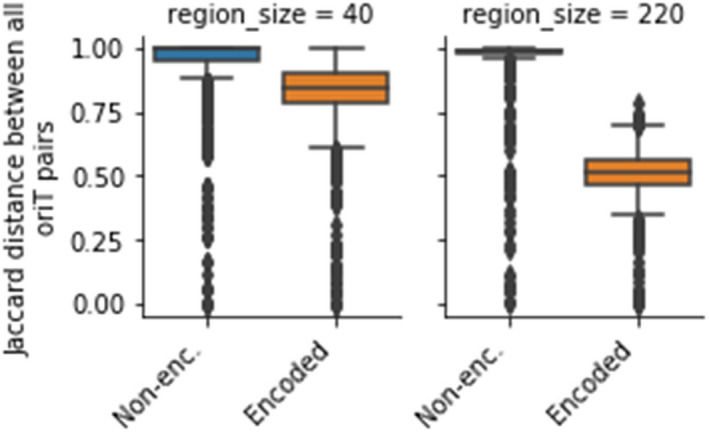
Distribution of pairwise Jaccard distances between *oriT*s, using structurally encoded k‐mers (Methods M2) or non‐encoded nucleotide k‐mers, with the subsets of different *oriT* sizes.

**FIGURE A3:**
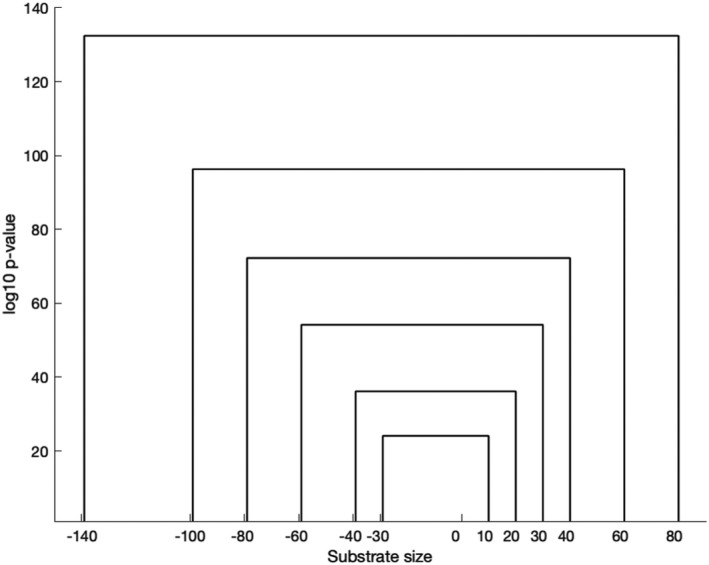
Schematic diagram of the estimated maximum statistical depth achievable with different sequence lengths, where an over 1e100‐fold difference is observed between the 40 bp and 220 bp sizes of *oriT*.

**FIGURE A4:**
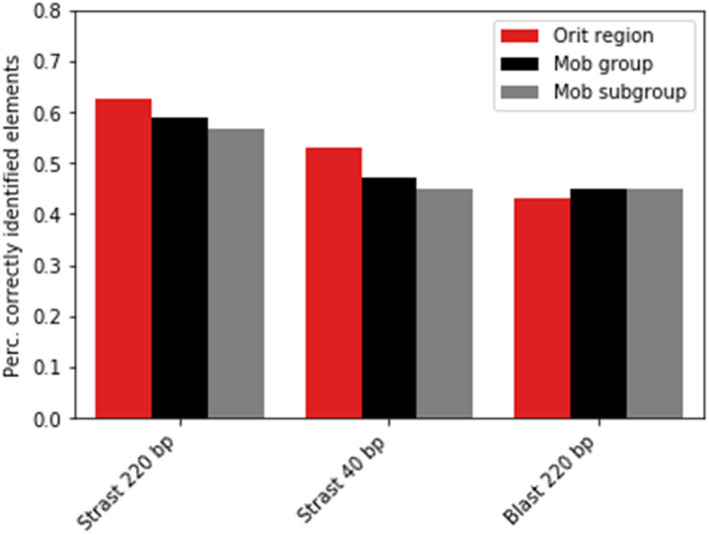
Percentage of correctly identified elements with the structural alignment algorithm (Strast) using the Mob‐typed testing dataset 1 with 220 and 40 bp query regions sizes and Blast with 220 bp region sizes.

**FIGURE A5:**
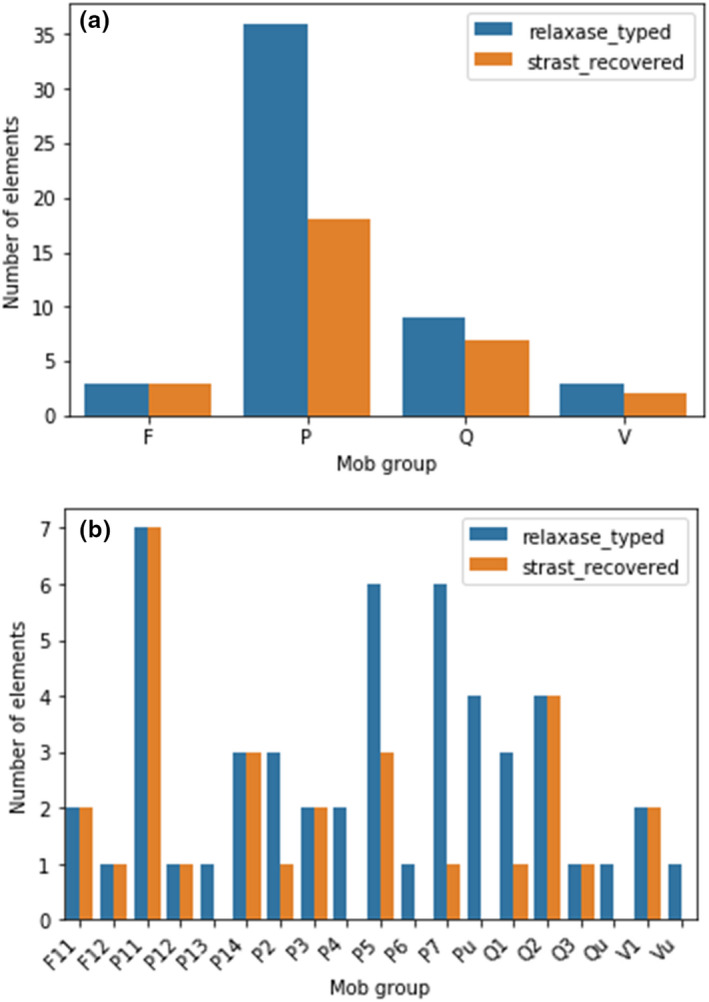
The number of recovered elements across (a) 4 Mob groups and (b) 19 Mob subgroups in the Mob‐typed testing dataset 1.

**FIGURE A6:**
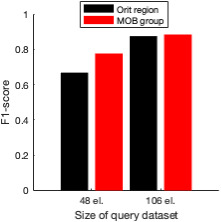
The effect of query dataset size on the performance of the structural alignment algorithm, where a diluted set of 48 elements was compared to the full query dataset.

**FIGURE A7:**
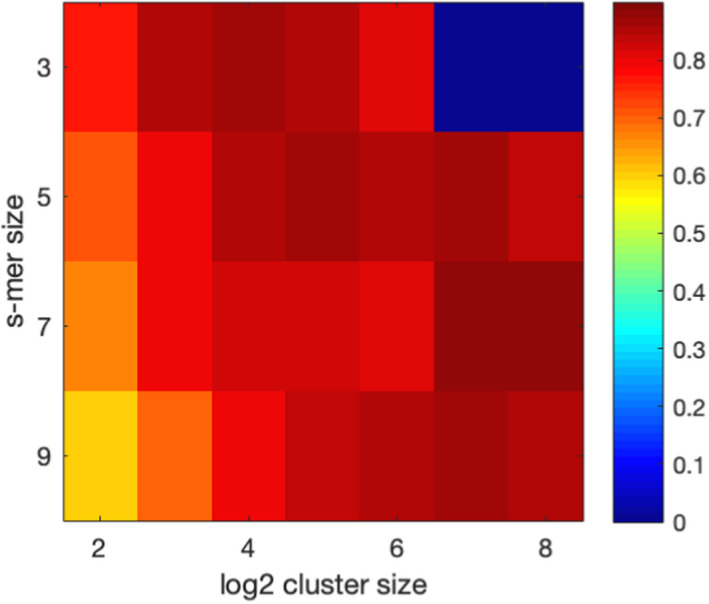
The effect of s‐mer size and number of clusters on the combined F1‐score of locating *oriT*s and Mob typing with the structural alignment algorithm. The combination of s‐mer size 7 and the number of clusters 2^7 (128) resulted in the best performance.

To compute DNA structural representations, 64 models of physicochemical and conformational DNA properties important for protein–DNA interactions, such as those occurring in *oriT* regions, were compiled (Table A1‐3). Next, to obtain the precomputed parameters for structurally defined groups of k‐mers, termed s‐mers (Figure A1‐1b), structural properties of all permutations of k‐mers of size *s* = 7 bp (3 neighboring regions around a specific nucleotide) were computed, after which dimensionality reduction and clustering were performed. Dimensionality reduction was performed using principal component analysis (PCA), and the number of used principal components was 18 (out of 64) to capture over 0.99 of the data variance. The k‐means clustering algorithm was used (MATLAB), where the number of clusters *k* was 128, and clusters with the lowest total sum of distances were chosen from 10 runs of up to 1000 iterations at default settings. The s‐mer size *s* and number of clusters *k* were chosen by comparing the algorithm performance using *s* = {3, 5, 7, 9} and *k* = {4, 8, 16, 32, 128, 256} (Zrimec, 2020), respectively (Figure A1‐7). Finally, the structural representation of a DNA sequence is obtained by encoding its k‐mers into s‐mers (Figure A1‐1b), where the length of the structural representation equals the length of the nucleotide sequence minus the leftover nucleotides at the borders (3 bp) due to the neighboring nucleotides in s‐mers.

The s‐distance between two DNA substrates was the sum of squared Euclidean distances between the cluster centroids of all equally positioned s‐mers in their structural representations of length *n*,

(1) $$s‐\text{distance}\;=\;\sum_{i=1}^{n}d(C1i,C2i)2,$$

where *C_ni_* = *c_n1_*, *c_n2_*, …, *c_nk_* are the cluster centroids of the s‐mer at position *i* of the first and second sequences, respectively. For algorithmic efficiency, the distances between all s‐mers were precomputed and stored in a distance matrix. The p‐distance was equal to the Hamming distance corrected for sequence length. The Jaccard distance between two DNA sequences was defined as the intersection over the union of sets of either their unique k‐mers, with nucleotide sequence representation, or s‐mers, with structural representation, respectively.

The performance of the alignment algorithm for typing *oriT*s in target sequences was tested by evaluating the correctness of both (a) *oriT* and *nic* location finding to within ±1 bp (Francia et al., 2004) and (b) typing of Mob groups and subgroups. Query region lengths of 220 bp and 40 bp, spanning whole *oriT* regions, and shorter 40 bp relaxase‐binding substrates (Figure 1a: −30 to +10 bo around the *nic* site), respectively, were assessed in both types of tests. For comparison to traditional non‐encoded sequence‐based algorithms, blastn v2.2.24 (Altschul et al., 1990; www.ncbi.com) was used with default settings (word size = 11, expectation threshold = 10, nucleic match/mismatch score = 2/−3, gap opening/extension costs = 5/2), where the same query and target data as with Strast were used to obtain alignment hits. The specific capability of Strast for locating *oriT* and *nic* regions was compared against the tool OriTfinder (Li et al., 2018; https://bioinfo-mml.sjtu.edu.cn/oriTfinder/), where the web‐based version was used with default settings (Blast *E*‐value = 0.01) by uploading fasta files of the target sequences and relying on the built‐in query sequences.

### 2.3 M3. Statistical analysis and machine learning metrics

The F‐test was performed using PERMANOVA (Anderson, 2001) with sequence bootstraps. The statistical significance of s‐distance scores was evaluated using permutational tests, where bootstrap resampling (n_bootstraps =1e6 per sequence) of randomly selected query *oriT* sequences (n_seq = 10) was used to estimate the s‐distance scores at different *p*‐value cutoffs (from 1e‐6 to 1e‐1). Next, to obtain a mapping function of s‐distance to permutational *p*‐values in the whole range of 1e‐132 to 1e‐1 (Figure A1‐1d), the least‐squares curve fitting to a second‐order polynomial function was performed, where the theoretical limit of ~1e‐132 was set to correspond to an s‐distance of 0. For additional statistical hypothesis testing, the Python package Scipy v1.1.0 was used with default settings.

The following machine learning performance metrics were used to assess alignment algorithm performance: Precision, Recall/Sensitivity, Specificity, Accuracy, F1‐score, and Matthews correlation coefficient (Table A1‐6). To calculate these metrics, true‐ and false‐positive and true‐ and false‐negative counts were obtained from the alignment tests (Methods M2) by considering only the most significant hit per alignment. A true‐ or false‐positive value was assigned if the result was above a specified significance cutoff and corresponded or did not correspond, respectively, to the known value (*nic* location, Mob group, or subgroup), and alternatively, a false‐ or true‐negative value was assigned to results below the significance cutoff that corresponded or did not correspond, respectively, to the known value.

### 2.4 M4. Analysis of alignment hits

The newly uncovered regions were analyzed by comparing the features of the *oriT* alignment hits with those of the query dataset, which included sequence properties and inverted repeats. Sequence homology analysis involved (a) calculation of the sequence homologies of the *oriT* query dataset within each Mob group (b) calculation of sequence homologies between each *oriT* hit and its closest‐associated query *oriT* region, (c) comparison of the sequence homologies of the *oriT* query and alignment hit datasets, across the different sized *oriT* subregions. *OriT* hits with sequences of their relaxase‐binding 40 bp subregions that deviated below 60% seq. homology from their query counterparts were removed. Sequence homology was calculated with the *ratio* function (python‐Levenshtein package v0.12), where it equaled the Levenshtein (edit) distance divided by the length of the sequence. Similarly, analysis of the inverted repeats (IRs) involved computation of imperfect IRs in both the *oriT* query and alignment hit datasets, and *oriT* hits lacking IRs similar to those in the query set were removed. The MATLAB package detectIR v2016‐01‐19 (Ye et al., 2014) was used with IR size limits of (6, 15) bp and containing at most 2 mismatches. From the initially identified 20,255 *oriT* hits, 11,497 (57%) were retained (Dataset S3: https://github.com/JanZrimec/oriT-Strast/blob/master/data/Dataset_S3.csv).

### 2.5 M5. Simulations of plasmid mobility

To estimate the results that would be obtained with a larger *oriT* query dataset, the following procedure was applied. The *oriT* alignment results with the dataset of 4602 target plasmids were diluted according to 10‐fold dilutions of the 102 query regions used to identify the hits (10 repetitions were used). Least‐squares curve fitting was performed (Python package Scipy v1.1.0) using a linear function and the dataset dilutions—specifically between the size of the query *oriT* dataset and the variables corresponding to the numbers of *oriT* hits, putative mobile plasmids, putative mobile plasmid‐carrying host species, and overlap with relaxase‐typed plasmids.

### 2.6 M6. Network analysis

To study the co‐occurrence of different *oriT* regions or Mob groups as nodes, shared across the putative multi‐*oriT* plasmids as edges, an undirected multi‐edged graph was constructed. The graph contained a total of 79,004 connections and the number of unique *oriT* nodes was 102 since each *oriT* hit was characterized by its closest‐associated query *oriT*.

To study the potential for plasmid transfer between different habitats, host species of the *oriT* alignment results within the subset of multi‐*oriT* plasmids were mapped across 9 habitat supertypes (Table A3‐4) according to published data on environmental (Pignatelli et al., 2009) and human microbiomes (Dewhirst et al., 2010; Escapa et al., 2018; Forster et al., 2016; Human Microbiome Project Consortium, 2012; Lloyd‐Price et al., 2017). This retained 43% (227 of 532) of the unique species carrying multi‐*oriT* plasmids, where habitat sizes reflected those of the full habitat dataset (according to the number of unique species, on average 939 species) but were on average eightfold smaller (on average 119 species) varying less than 22% around this value. The habitat taxonomy was further expanded to include human commensal and pathogen types (Human Microbiome Project Consortium, 2012) as well as tissue subtypes (Pignatelli et al., 2009). Next, a directed graph representation of habitat nodes connected by potential plasmid transfers as edges was constructed, where habitats of donor hosts carrying the putative mobile plasmids (outbound connections) connected to habitats of potential acceptor hosts deduced from the query *oriT*s (inbound connections). The network comprised 141,395 connected habitat node pairs, with a total of 1,600,978 plasmid connections between the habitats.

For network analysis, the Python package NetworkX v2.2 was used. For typing antimicrobial resistance genes in the plasmids, the webserver version of ResFinder v3.2 (Zankari et al., 2012) was used with default settings.

### 2.7 M7. Software

MATLAB v2018 (www.mathworks.com) and Python v3.6 (www.python.org) were used.

## 3 RESULTS

### 3.1 Structural alignment algorithm improves *oriT* typing performance

A DNA alignment algorithm performs multiple comparisons between a query and a target sequence by evaluating a distance function. We thus developed a structural distance function, termed s‐distance (Figure A1‐1, Methods M2), which was based on encoding the DNA sequences into structural representations and defined as the sum of squared Euclidean distances between two DNA structural representations. This enabled the comparison between structurally encoded *oriT*s and non‐encoded ones, where the ungapped p‐distance was used. For this comparison, a balanced dataset of 64 *oriT* regions from 4 Mob groups F, P, Q, and V (Zrimec & Lapanje, 2018) was used (Methods M1), where region sizes were varied stepwise to cover a single relaxase enzymatic site of 40 bp up to the whole *oriT* region of 220 bp containing multiple binding sites (Figure 1a). Furthermore, the comparison included discrimination of both (a) positive *oriT*s (aligned to the *nic* site) from negative non‐*oriT* sequences and (b) Mob groups. With *oriT* structural representations, significant (ANOVA *p* < 1e‐4) discrimination of both positive/negative and Mob groups was achieved in the whole *oriT* size range (Figure 1b,c), compared to the non‐encoded nucleotide sequences, where results were significant (ANOVA *p* < .05) only with *oriT* regions equal to or shorter than 120 bp. This was corroborated with the Jaccard distance, which significantly (ranksum *p *< 1e‐16) decreased by 40% with increasing *oriT* region size when using structurally encoded k‐mers (Methods M2, Figure A1‐2), whereas it increased with nucleotide k‐mers (ranksum *p* < 1e‐9). The results suggested that our structural encoding approach leveraged the chemical information in longer query regions and could thus improve multiple sequence comparisons with alignments by increasing the statistical depth (Figure A1‐3).

We next prototyped an alignment framework (Figure 1d) that employed the s‐distance measure to find target hits to query *oriT*s, where *p*‐values were obtained via permutation tests (Figure A1‐1, Methods M2). A query dataset of 106 query *oriT* regions from the 4 Mob groups F, P, Q, and V was compiled as well as two testing datasets with experimentally determined *oriT* regions comprising altogether 64 plasmids, 51 Mob‐typed (Francia et al., 2004; Garcillán‐Barcia et al., 2009) and 13 non‐Mob‐typed (Li et al., 2018), respectively (Table A1‐1, Methods M1). The algorithm's performance was first tested by assessing the *oriT* location and Mob type of the highest‐scoring alignment hits using the testing dataset of Mob‐typed plasmids (Methods M2). By using full‐length 220 bp query regions, on average, 19% more significant (permutation test *p* < 1e‐13) *oriT* hits were recovered, and 25% more Mob groups were correctly predicted compared to using a 40 bp query size (Figure 1e, Figure A1‐4). This corroborated that the use of longer queries indeed led to improved algorithm performance (Figure 1b,c). Furthermore, compared to Blast (Altschul et al., 1990), our approach uncovered on average 45% more significant (permutation test *p* < 1e‐13) *oriT* hits and correctly predicted 30% more Mob groups (Figure 1e, Figure A1‐4). By analyzing machine learning metrics to better understand the algorithm's performance (Methods M3), a marked 43% increase was observed with *Recall* at a relatively constant *Precision* and *Specificity* (Figure 1f, Table A1‐2), which corresponded to recovering a larger amount of the correct *oriT*s (Figure 1e). The new algorithm thus correctly located and Mob‐typed on average 61% of *oriT*s in the testing dataset (Figure A1‐5).

The capability of the algorithm to identify specifically *nic* sites was further validated using the testing dataset of plasmids that were not Mob‐typed (Li et al., 2018; Table A1‐3). Out of 13 such plasmids with 14 *oriT* sites, it correctly identified (permutational test *p* < 1e‐12) 6 *oriT* regions in 5 plasmids with 100% sequence identity and aligned to within ±1 bp of the *nic* sites (Francia et al., 2004; see Table A1‐3). In contrast, the tool OriTfinder (Li et al., 2018) was able to correctly identify the approximate locations of 10 *oriT* regions; however, it correctly determined the *nic* locations in only 5 of these *oriT*s, to within ±1 bp (Table A1‐4). The results indicated that due to the lack of diversity in the query dataset our algorithm altogether missed certain *oriT*s in the testing datasets, which was also confirmed by using smaller query datasets that lowered the algorithm's performance especially for locating *oriT* regions (Figure A1‐6). Nevertheless, despite the limited *oriT* data availability, the results experimentally verified the algorithm's capacity for *oriT* typing.

### 3.2 *OriT* typing reveals a twofold increase in the number of putative mobile plasmids

**FIGURE 2:**
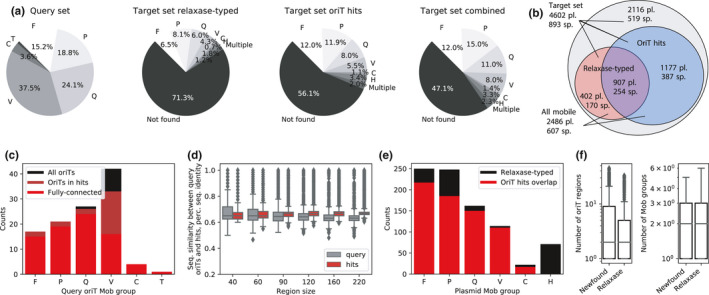
*OriT* typing reveals a twofold increase in the number of putative mobile plasmids. (a) Distribution of Mob groups across the query *oriT* and target plasmid datasets, where the latter was analyzed using either relaxase typing ^51^, structural alignment‐typing, or a combination of both methods. (b) Venn diagram of the number of plasmids (pl.) and plasmid‐carrying host species (sp.) in the whole target plasmid dataset and the separate subsets uncovered to be putatively mobile by either structural alignment or relaxase typing. (c) Distribution of Mob groups in the whole query *oriT* dataset and in the query subsets that returned alignment hits or were present in the fully connected putative *oriT* co‐occurrence network (see Results chapter 3). (d) Average sequence identities across Mob groups with different *oriT* size subsets, calculated pairwise between all query *oriT*s and between each *oriT* hit and its closest‐associated query *oriT*s within a Mob group. (e) Distribution of Mob groups across relaxase‐typed and structural alignment‐typed plasmids. (f) Distributions of amounts of putative *oriT* regions and Mob groups across the structural alignment‐typed (newfound) and relaxase‐typed plasmids.

The structural alignment algorithm was used to explore the diversity of *oriT* regions in natural plasmids. To cover all available *oriT* regions, the query dataset was expanded to 112 unique *oriT*s from 6 Mob groups that included, besides *oriT*s from the major Mob groups F, P, Q, and V, also 3.6% and 0.9% of *oriT*s from groups C and T, respectively (Figure 2a, Methods M1). The query dataset covered 59 unique host species with the majority (88.4%) from the phyla *Proteobacteria* and *Firmicutes* (Figure A2‐1). The target dataset comprised 4602 natural plasmids with Mob groups determined by relaxase amino acid homology analysis (Shintani et al., 2015; Figure 2b). Here, 28.7% of plasmids were putatively mobile (1307 plasmids that contained 1377 distinct relaxases), with the highest represented Mob groups F, P, Q, and V (Figure 2a). The target dataset contained 893 distinct host species from 22 distinct phyla, with the putative mobile plasmids harbored by 40% of the distinct species with 88.1% from the phyla *Proteobacteria* and *Firmicutes* (Figure A2‐1).

Based on the 1377 distinct relaxases found in 1309 plasmids in the target dataset (Figure 2b: 28.4% of target dataset), at least a similar amount of *oriT* regions can be expected. However, with our alignment method, we identified 11,497 significant (*q*‐value <1e‐8, *E*‐value <0.01) *oriT* hits present in 69.2% (907) of the relaxase carrying plasmids as well as in an additional 1177 plasmids, in which a relaxase had not been found (Methods M4). The *oriT* hits were uncovered with 91% (102/112) of the query regions and covered all Mob groups and subgroups (Figure 2c). They contained sequence features, including sequence homology and inverted repeats (IR) that facilitate relaxase recognition and binding (Zrimec & Lapanje, 2018), similar to those of the query regions and in accordance with published findings showing that relaxases can function with relaxed specificity on non‐cognate *oriT*s with ~60% sequence homology (Fernández‐López et al., 2013; Kishida et al., 2017; O’Brien et al., 2015). Indeed, sequence homologies between an *oriT* hit and its closest‐associated query *oriT* region were above 60% within all the 40 bp relaxase‐binding sites and with the majority (>99.6%) of the larger sized subregions (Figure 2d). Median sequence homologies were well above the ones of the query dataset, where all pairwise seq. homologies within each Mob group were measured (Figure 2d, Methods M4). Furthermore, all the different sizes of *oriT* subregions were strongly correlated (Pearson's *r* > 0.730, *p* < 1e‐16, Table A2‐1) between each other as well as to the *oriT* structural similarity, s‐distance (Pearson's *r* > 0.817, *p* < 1e‐16, Figure A2‐2). Following the analysis of IRs in the query *oriT*s, where in the 60 bp upstream of *nic* an IR of at least 6 bp could be identified (Figure A2‐3A: avg. size was 10 bp, Methods M4), in the *oriT* hits all but 6 *oriT*s (0.05%) carried IRs with similar properties (Figure A2‐3B: at least 6 bp with an avg. size of 10 bp). Finally, we explored if the putative *oriT*s were located in any specific coding or non‐coding regions in the plasmids by obtaining and analyzing the CDS records of each plasmid (Methods M3). Indeed, a significant (Fisher's exact test *p* < 1e‐16) 4.6‐fold increase of *oriT* presence was observed in non‐coding areas and a twofold decrease in coding ones, as well as a significant (Fisher's exact test *p* < 1e‐16) enrichment in genes related to horizontal mobility, namely conjugation, transposition, and integration (Table A2‐2).

The putative *oriT* regions combined with the previously typed relaxases resulted in a total of 2486 putative mobile plasmids (Figure 2b: 54% of target dataset), which represented a 1.9‐fold increase compared to the initial relaxase typing. Similarly, a 1.7‐fold increase in the number of putative mobile plasmid‐carrying host species was identified, when comparing species from the whole set of putative mobile plasmids (Figure 2b: 607 out of 893 species, 58.2%) with the previous relaxase‐typed ones (356 species, 39.9%). This also corresponded to a 1.4‐fold increase in the number of distinct Phyla, with putative mobile plasmids representing 19 out of the 23 Phyla compared to 14 with relaxase typing (Figure A2‐1). Furthermore, out of the 907 plasmids where both *oriT*s and relaxases were identified, the same Mob group, indicating that the *oriT* was cognate to the relaxase, was identified in 75% of cases (Figure 2e). In the remaining 25% of these plasmids, the *oriT* hits could have been secondary *oriT*s (Becker & Meyer, 2003; Parker et al., 2005) or corresponded to either unknown or *in trans* acting (O’Brien et al., 2015) relaxases. The distribution of the *oriT*‐identified Mob groups was found to be comparable to the one expected according to relaxase typing (Figure 2e). Multiple putative *oriT* regions were identified in over 63% of both the relaxase‐typed and untyped plasmids, where on average, 2 *oriT*s from 2 different Mob groups were identified per plasmid (Figure 2f). This supported the notion that, besides secondary and *in trans oriT*s, the untyped plasmids possibly carried unidentified relaxases (Coluzzi et al., 2017; Garcillán‐Barcia et al., 2009; Guzmán‐Herrador & Llosa, 2019; Ramachandran et al., 2017; Soler et al., 2019; Wisniewski et al., 2016).

Since the number of query *oriT*s was the limiting factor in our analysis of plasmid mobility, we explored what effect a larger query dataset could have on the findings. Briefly, the use of a larger query dataset was simulated by performing curve fitting on results obtained with 10 repetitions of random 10‐fold dilutions of the present dataset (Methods M5). An approximate linear rule was observed between the size of the query dataset and the number of uncovered *oriT*s, as each order of magnitude increase in *oriT* hits required likewise an order of magnitude larger query dataset (Figure A2‐4A: e.g., 1e5 hits would require a query set of ~975 *oriT*s). Consequently, with each order of magnitude increase of the size of the query dataset, approximately 1500 more putative mobile plasmids (Figure A2‐4b: starting from an initial value of 500 with 10 *oriT* queries) and 250 more putative mobile plasmid‐carrying host species were uncovered (Figure A2‐4c). Additionally, to achieve a full overlap with the relaxase‐typed plasmids, a considerably larger query dataset than is currently available would be required, comprising 415 *oriT*s (95% lower and upper bounds were 328 and 532, respectively, Figure A2‐4d). The demonstrated limitations of the query data suggest that the present published results (Shintani et al., 2015; Smillie et al., 2010) and our findings might still be an underestimation of the true plasmid mobility present in nature.

### 3.3 The presence of multiple putative *oriT* regions might aid plasmid transfer between habitats

**FIGURE 3:**
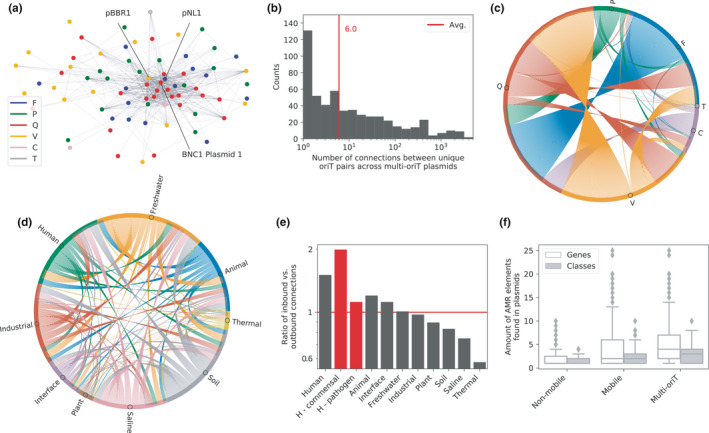
The presence of multiple putative *oriT* regions might aid plasmid transfer between habitats. (a) Undirected multi‐edged graph of *oriT* (nodes) co‐occurrence across plasmids (edges). The most frequently co‐occurring *oriT*s from plasmids pBBR1, pNL1, and BNC1 Plasmid 1 are marked. The putative *oriT*s were represented by their closest‐associated query *oriT*s. (b) Distribution of the number of unique *oriT* co‐occurrences across the multi‐*oriT* plasmids. (c) Undirected graph of Mob group co‐occurrence in multi‐*oriT* plasmids based on *oriT* co‐occurrence. (d) Directed graph depicting the hypothetical connectivity of habitats (nodes) based on potential plasmid transfers (edges). Outbound connections are based on habitats of donor hosts carrying the putative mobile plasmids, and inbound connections are habitats of potential acceptor hosts deduced from the query *oriT*s. (e) The ratio of inbound vs. outbound connections across different habitats, where human commensal and pathogen microbiomes are highlighted in red. (f) Amount of AMR genes and classes found in non‐mobile and putative mobile plasmids.

A large part of the newly uncovered *oriT*s were additional regions to the primary ones that corresponded to the plasmid cognate relaxases (Figure 2f), resulting in 1331 multi‐*oriT* plasmids (54% of the putative mobile plasmids) that carried on average 5 *oriT* hits (Figure A3‐1). First, we analyzed the co‐occurrence network between the different putative *oriT* regions (nodes), when they were carried by the same multi‐*oriT* plasmids (edges; Figure 3a, Methods M6). Since each *oriT* hit was characterized only by its closest‐associated query *oriT*, the actual *oriT* node diversity was limited to the 102 query *oriT*s that returned hits (Figure 2e). The network contained 552 unique co‐occurring *oriT* node pairs (Figure 3a), with an average of 6 co‐occurrences and up to 3528 co‐occurrences per *oriT* node pair (Figure 3b). A fully connected component was found, which comprised of 76 *oriT* nodes (75% of all *oriT*s, Figure 3a) that proportionally represented all 6 Mob groups except Mob V (Figure 2c: only 38% of Mob V in the fully connected subgraph). Further network analysis showed that this *oriT* network obeys the laws of natural biological scale‐free networks, with possibly a hierarchical topology (Barabási & Oltvai, 2004; Figure A3‐2). Indeed, specific *oriT* regions acted as hubs and co‐occurred with multiple other regions across the Mob groups (Figure 3a), with the most highly connected pNL1‐, BNC1 Plasmid 1‐ and pBBR1‐like *oriT*s from Mob F, Q, and V, respectively, co‐occurring with over 50 unique *oriT*s from all 6 Mob groups (Table A3‐1). We next investigated the co‐occurrence of Mob groups (Figure 3c, Methods M6) and measured a 75‐fold increase in the amount of Mob group co‐occurrences compared to relaxase typing. Over 90% of the multi‐*oriT* plasmids contained on average 2 unique Mob groups and 3 unique Mob subgroups (Figure A3‐1). The most frequently co‐occurring Mob groups were F, Q, and V, where 35,062 co‐occurrences were measured within Mob Q, 15,081 between Q and F, and 12,637 between Q and V (Figure 3c, Table A3‐2). with the main co‐occurring subgroups Mob Qu with Q2, Fu, V2 (Table A3‐3).

The above results suggested that each multi‐*oriT* plasmid might contain the initial means for mobilization by conjugation systems belonging to different MOB groups, and could, under specific conditions, connect multiple different plasmid host species either transiently, such as via transient transfer hosts (Klümper et al., 2015; Shintani et al., 2014), or via replicative hosts (Garcillán‐Barcia et al., 2011; Zrimec & Lapanje, 2018). To investigate what potential effect this could have on connecting different host species across different environmental habitats, and whether it could facilitate the spread of AMR genes to humans, a hypothetical network was built, where habitats (nodes) were inferred from the putative mobile plasmid‐carrying host species and connected via potential plasmid transfers (edges; Figure 3d, Methods M6). On average, each plasmid was present in 6 habitats and had access to 5 unique host species as well as 6 habitats (Figure A3‐3). The network was directed (Figure 3d), and the numbers of outbound and inbound connections, though differing across the habitat types (Figure A3‐4), were strongly correlated (Pearson's *r* = 0.894, *p* < 2e‐3). However, despite this, a marked increase was observed in the ratio of inbound vs. outbound connections in the human and animal habitats compared to the others (Figure 3e: 61% and 29% increase, respectively). Importantly, a twofold higher amount of inbound vs. outbound connections was measured with human commensals (Figure 3e), whereas human pathogens displayed a mere 11% increase. Further analysis of the potential transfer only within the human system showed an approximately equal rate of outbound and inbound connections. This is in accordance with previous observations showing that commensal bacteria can act as an interface for horizontal uptake of genes from the environment, which they might then disseminate to the pathogens within the human body (Forsberg et al., 2012; Marshall et al., 2009; Sommer et al., 2010).

Interestingly, among the highest amount of *oriT*s per plasmid was found in the industrial habitat, which included food production and water treatment facilities, and the animal habitat, both known to harbor resistance (Bengtsson‐Palme et al., 2018; Founou et al., 2016; Hu et al., 2016; Figure A3‐5). We, therefore, identified AMR genes in the plasmids (Methods M6), finding a moderate positive correlation (Pearson's *r* = 0.462, *p* < 1e‐16) between the number of putative *oriT*s and the number of identified resistance genes in a plasmid (Figure 3f). Thus, 33% of the multi‐*oriT* plasmids carried on average 4 genes from 3 different AMR classes (Figure 3f). When viewed from the perspective of the hypothetical cross‐habitat transfer network (Figure 3d), the number of inbound connections of resistance genes from the environment to the human microbiota indicated that the most abundant flow of genes corresponded to the oldest and most widely used classes of antibiotics, for which also resistance is most developed and widespread (Hutchings et al., 2019; Figure A3‐6). In this case, the number of inbound connections to pathogens surpassed that of commensals by almost 20%, possibly since AMR transfer routes might serve a different portion of microbes compared to plasmid transfer in general (Hu et al., 2016; Pal et al., 2016).

## 4 DISCUSSION

Here, the potential for horizontal transfer of natural plasmids is explored by attempting to identify all conjugative DNA origin‐of‐transfer substrates coded within them. By prototyping a structural alignment approach to find and characterize *oriT* regions across plasmids (Figure 1d), an almost eightfold larger amount of putative *oriT*s is found than can be expected according to relaxase typing (Figure 2b). Analysis of these regions indicates that the number of transferable plasmids could be as much as twofold higher and span almost twofold more host species than is currently known (Figure 2b). Consequently, over half of all putative mobile plasmids might contain the initial means for mobilization by conjugation systems belonging to different MOB groups and subgroups (Figure 3c), potentially linking multiple host ranges that are currently understood to be confined (Garcillán‐Barcia et al., 2011; Zrimec & Lapanje, 2018; Figure 3d).

Our *oriT* typing procedure is a result of rationally expanding DNA alignment algorithms to incorporate enzymatically relevant properties of the *oriT* substrates (Figure 1a), where the conservation of structural properties is detected across the whole 220 bp region compared to mere ~40 bp of the nucleotide sequence in the core relaxase‐binding site (Zrimec & Lapanje, 2018; Figure 1c,d). By allowing the use of at least twofold longer query sequences, structural alignment achieves a much larger statistical depth than sequence alignment (Figure A1‐3), which means that *oriT*s can be efficiently sought across whole plasmids instead of just the vicinity of relaxases (Li et al., 2018; Figure 1e). Since, due to the nature of the conserved structural properties, each enzymatic substrate corresponds to multiple possible sequence variants, the benefit of the DNA structural encoding is that it exposes these sequence variants by accessing the search space of the enzymatic co‐evolutionary constraints (i.e., DNA structural background; Zrimec, 2020; Zrimec & Lapanje, 2018; Figures 1a and 2d). The identified candidate *oriT* regions serve as starting points that can be further verified by typing other known molecular features (O’Brien et al., 2015), such as inverted repeats (Lanka & Wilkins, 1995; Williams & Schildbach, 2006; Figure A2‐4) and nucleotide sequence properties of the core enzymatic binding (Carballeira et al., 2014; Williams & Schildbach, 2007) and nicking sites (Francia et al., 2004; Vedantam et al., 2006; Figure 2d). Compared to established tools like OriTfinder, our method performs similarly, though with some complementarity (Tables A1‐3 and A1‐4), suggesting that it is a useful complement to the existing methods. However, its main advantage is the capability to determine Mob groups from mere *oriT* regions (accuracy >90%, Table A1‐2) without the requirement for relaxase typing, which also enables typing *oriT*s in plasmids without a (known) relaxase (da Cruz Campos et al., 2019).

**FIGURE A8:**
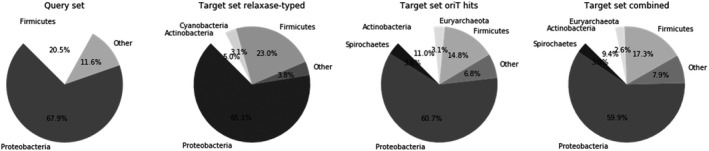
Distributions of phyla in the query dataset as well as in the target dataset obtained by relaxase and structural alignment‐typing.

**FIGURE A9:**
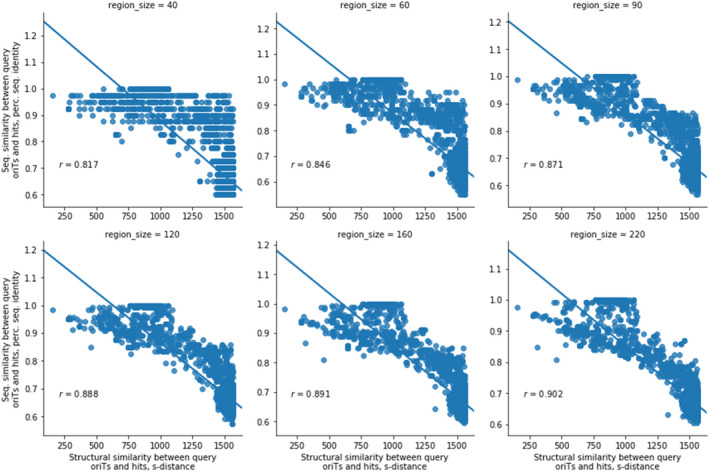
Correlation analysis between the sequence homology and structural similarities (s‐distance) among *oriT* hits and their closest‐associated query sequences. All *p*‐values were below 1e‐16.

Besides uncovering the majority of expected *oriT* regions of known cognate relaxases (Figure 2e), almost ⅔ of the putative *oriT*‐bearing plasmids carry multiple *oriT*s (Figure 2f). These putative *oriT*s are frequently located where they are expected, in non‐coding regions and within genes related to horizontal mobility (conjugation, transposition, and integration; De La Cruz et al., 2010). However, the number of Mob groups and depth of enzymatic substrate diversity that could be analyzed within each group was constrained by the size of the query set of available *oriT* regions and *nic* sites (Garcillán‐Barcia et al., 2020; Figures A1‐9 and A2‐5). By simulating the availability of a larger set of query sequences, a linear relationship between the amount of uncovered and query *oriT*s indicates that our current sampling might still be an underestimation of the actual plasmid mobility in nature that could span all plasmids (Gillings, 2013; Smillie, et al., 2010; Figure 2i). The results of a twofold higher putative plasmid mobility compared to relaxase typing, with an almost similar increase in the amount of putative mobile plasmid‐bearing host species (Figure 2b), point to multiple possibilities that further undermine the paradigm of a one relaxase‐one *oriT* conjugative plasmid system spanning less than ⅓ of plasmids (Shintani et al., 2015): (a) a massive under‐identification of relaxase enzymes (Chandler et al., 2013; Smillie, et al., 2010), (b) relaxase promiscuity (Becker & Meyer, 2003; Guzmán‐Herrador & Llosa, 2019) and *oriT* evolutionary mechanisms (Parker et al., 2005) leading to many functional secondary *oriT*s, and (c) a system‐wide adoption of relaxase *in trans* mechanisms (Guzmán‐Herrador & Llosa, 2019; Ramsay et al., 2016).

Plasmids are vehicles for the transfer and long‐term storage of ‘common goods’ that include, besides AMR, also virulence, heavy metal resistance, and other genes (Bukowski et al., 2019). Based on the usefulness of this cargo, one can expect that the global plasmid transfer network possesses at least some properties of a robust fault‐tolerant system that would increase the guarantee for transfer and information storage (Gillings, 2013; Han et al., 2007). Furthermore, recent findings suggest that certain plasmids might be able to bypass key horizontal transfer barriers (Ben Maamar et al., 2020; Dolejska & Papagiannitsis, 2018; Malhotra‐Kumar et al., 2016; San Millan, 2018; Wang & Sun, 2015; Wintersdorff et al., 2016), including phylogenetic (Acman et al., 2020; Hu et al., 2016; Soucy et al., 2015), host range (defined by Mob and Inc/Rep groups, respectively; Garcillán‐Barcia et al., 2011; Orlek et al., 2017; Shintani et al., 2015; Zrimec & Lapanje, 2018) as well as ecological habitat constraints (Bengtsson‐Palme et al., 2018; Hu et al., 2016; Manaia, 2017; Thanner et al., 2016), for instance, in the transmission of AMR from environmental reservoirs to human hosts (Manaia, 2017; Mathers et al., 2015; Sun et al., 2019; Wintersdorff et al., 2016). To this end, the putative *oriT* network topology via the closest‐associated query *oriT*s is reminiscent of scale‐free and even hierarchical networks (Figure 3a) and thus displays robust fault‐tolerant properties. As sparsely connected nodes without many direct neighbors are linked to highly connected hubs, even in case of absence of a large number of nodes, the remaining ones are likely still well connected (Barabási & Oltvai, 2004; Seyed‐Allaei et al., 2006). Moreover, plasmids bearing multiple putative oriTs that could be mobilized by different conjugative systems (Figure 3c) possess at least the initial means that could enable them to transcend some of the horizontal transfer barriers (Gillings, 2013; Haaber et al., 2017; Siefert, 2009). In this view, one can hypothesize that certain conjugative transfer mechanisms and their corresponding hosts might act as transfer hubs that help to ensure the flow of genetic information among the different global microbiomes (Manaia, 2017; Perry & Wright, 2013; Tamminen et al., 2012). Interestingly, following these expected properties, the amount of identified plasmid‐borne AMR genes is found to be proportional to the assessment of putative plasmid mobility (Figure 3f and Figure A3‐6).

Care should be taken with interpretation of the hypothetical network of plasmid transfers between different hosts and ecological habitats (Figure 3d), due to key limitations in its analysis. Actual plasmid transfer is not dependent merely on the correct combinations of *oriT* and relaxase but constrained by additional genetic context due to plasmids being highly modular systems (Acman et al., 2020; Nishida, 2012; Shintani et al., 2015; Smillie, et al., 2010), which was not accounted for here. Nevertheless, the hypothetical network displays interesting properties, not at all different from ones that can be expected based on current knowledge (Haaber et al., 2017; Lopatkin et al., 2017; Manaia, 2017; Marshall et al., 2009). For instance, the considerably larger influx of plasmids to humans and animals compared to other environmental habitats (Figure 3d) might be a consequence of the increased amount of AMR transfers to these organisms (Bengtsson‐Palme et al., 2018; Dolejska & Papagiannitsis, 2018; Wintersdorff et al., 2016). In accordance with published findings (Forsberg et al., 2012; Forslund et al., 2013; Marshall et al., 2009), human commensals might act as the main interface for horizontal uptake of genes from the environment in general (Figure 3e), whereas the transfer of the specific widespread AMR genes might be more highly targeted at pathogens (Figure A3‐6). Despite the hypothetical nature of the network analysis based merely on first principles (Figure 3a,c,d), the potential increase in putative plasmid mobility that it shows could potentially be an important driver of the observed rapid resistance development in humans (Dolejska & Papagiannitsis, 2018; Manaia, 2017) and thus an important point of focus for further research as well as the development of prevention measures.

## CONFLICT OF INTEREST

None declared.

## AUTHOR CONTRIBUTION

**Jan Zrimec:** Conceptualization (equal); Data curation (equal); Formal analysis (equal); Funding acquisition (equal); Investigation (equal); Methodology (equal); Project administration (equal); Resources (equal); Software (equal); Supervision (equal); Validation (equal); Visualization (equal); Writing‐original draft (equal); Writing‐review & editing (equal).

## ETHICS STATEMENT

None required.

## Figures and Tables

**FIGURE A10:**
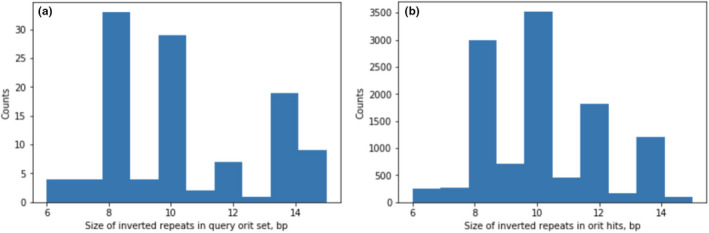
Distributions of sizes of inverted repeats identified in the (a) query dataset and (b) dataset of *oriT* hits.

**FIGURE A11:**
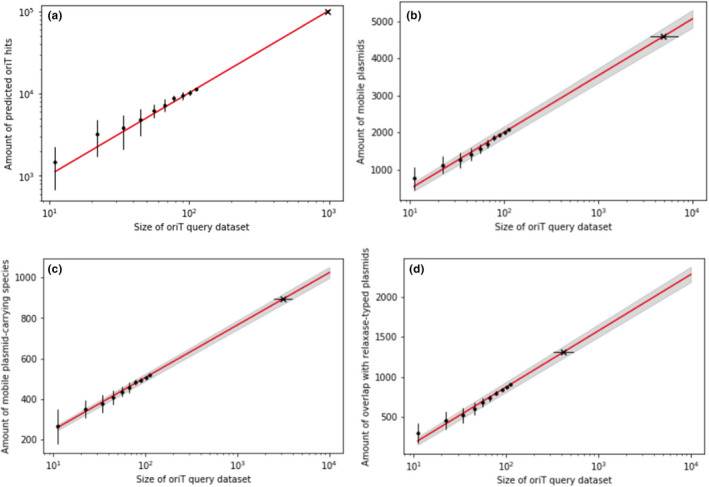
Simulated effect of the size of the query dataset on the (a) amount of uncovered *oriT* regions, (b) amount of putative mobile plasmids, (c) amount of putative mobile plasmid‐carrying host species, and (d) amount of overlap with relaxase‐typed plasmids. Red lines denote least‐squares fit, and gray areas denote 95% confidence intervals. Black dots and error bars denote 10 repetitions of 10‐fold dataset dilutions used for curve fitting. 'X' and vertical error bars denote predictions that mark the size of the query dataset required to recover (a) 1e5 *oriT* hits (query dataset of 975 *oriT*s with 95% lower and upper bounds within 0.07 of this value, respectively), (b) *OriT* regions spanning the whole target dataset of 4602 plasmids (query dataset of 4940 *oriT*s, 95% lower and upper bounds were 3548 and 7003, respectively), (c) the whole species diversity of the target dataset ‐ 893 unique species (query dataset of 3101 *oriT*s, 95% lower and upper bounds were 2491 and 3891), (d) a full overlap with the relaxase‐typed plasmids (query dataset of 415 *oriT*s, 95% lower and upper bounds were 328 and 532, respectively). Horizontal and vertical error bars denote 95 % confidence intervals.

**FIGURE A12:**
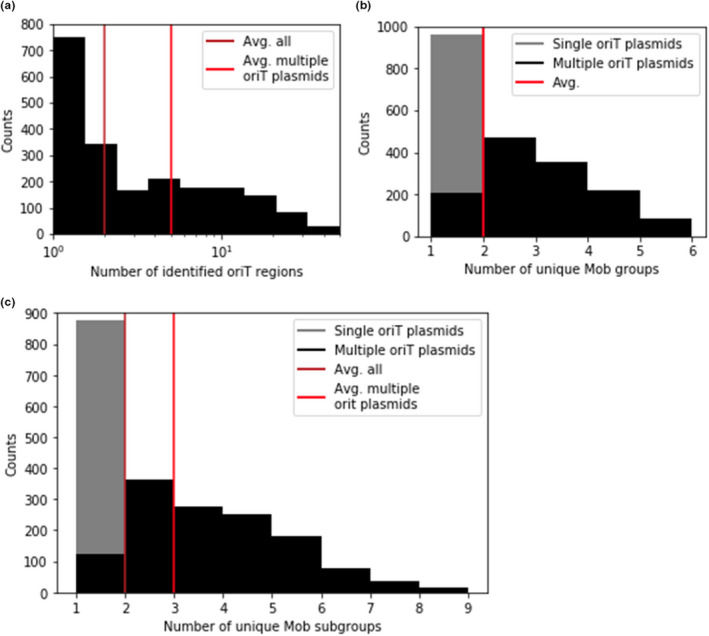
(a) Distribution of the amount of identified *oriT*s. Separate median averages are given for all mobile plasmids and those with multiple *oriT*s. (b) Distribution of the number of unique Mob groups in single and multiple *oriT* plasmids. Median averages for all mobile plasmids and those with multiple *oriT*s are the same. (c) Distribution of the number of unique Mob subgroups in single and multiple *oriT* plasmids. Separate median averages are given for all mobile plasmids and those with multiple *oriT*s.

**FIGURE A13:**
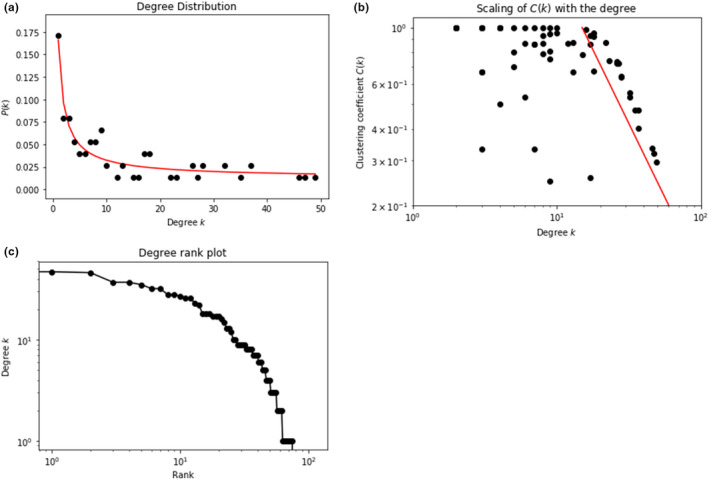
(a) Degree distribution with the power law function (x^−a^ b + c) fit to the data using least‐squares regression (red line). Exponents were 0.965, 0.147, and 0.012, respectively. The characteristics of networks with degree exponents below 2 are that they are highly dependent on hubs (Barabási & Oltvai, 2004) and that making new links between nodes is inexpensive Seyed‐Allaei et al. (2006). (b) Scaling of clustering coefficient with the degree of connectivity per node. The average clustering coefficient was 0.47. The red line denotes an approximate linear fit. (c) Degree rank plot.

**FIGURE A14:**
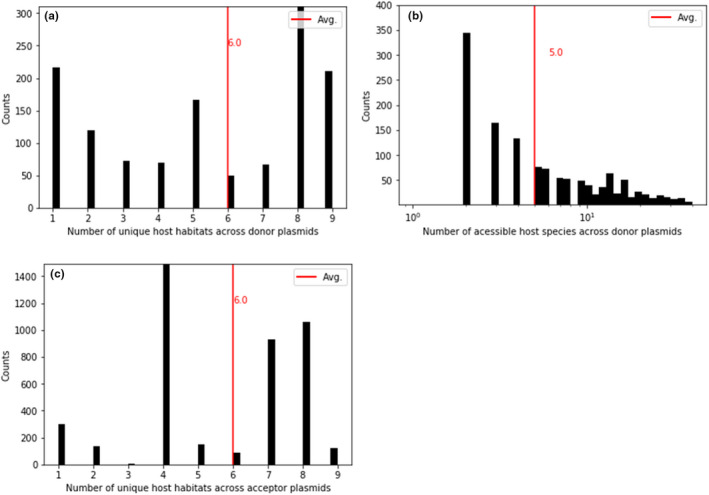
Properties of the horizontal transfer network. (a) Distribution of the number of unique host habitats per plasmid. (b) Distribution of the number of potentially accessible hosts of the multi‐*oriT* donor plasmids. (c) Distribution of the number of unique accessible host habitats via *oriT*s.

**FIGURE A15:**
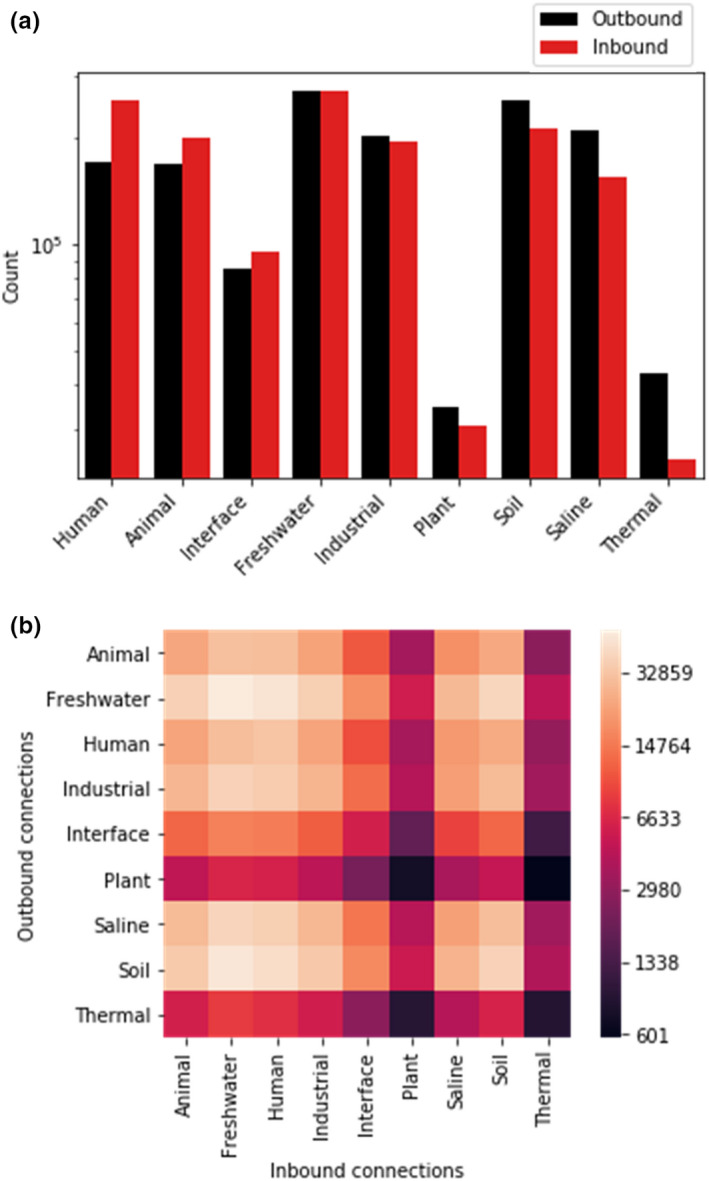
(a) The number of outbound and inbound connections across the different habitats. (b) The adjacency matrix of outbound vs. inbound connections across the different habitats.

**FIGURE A16:**
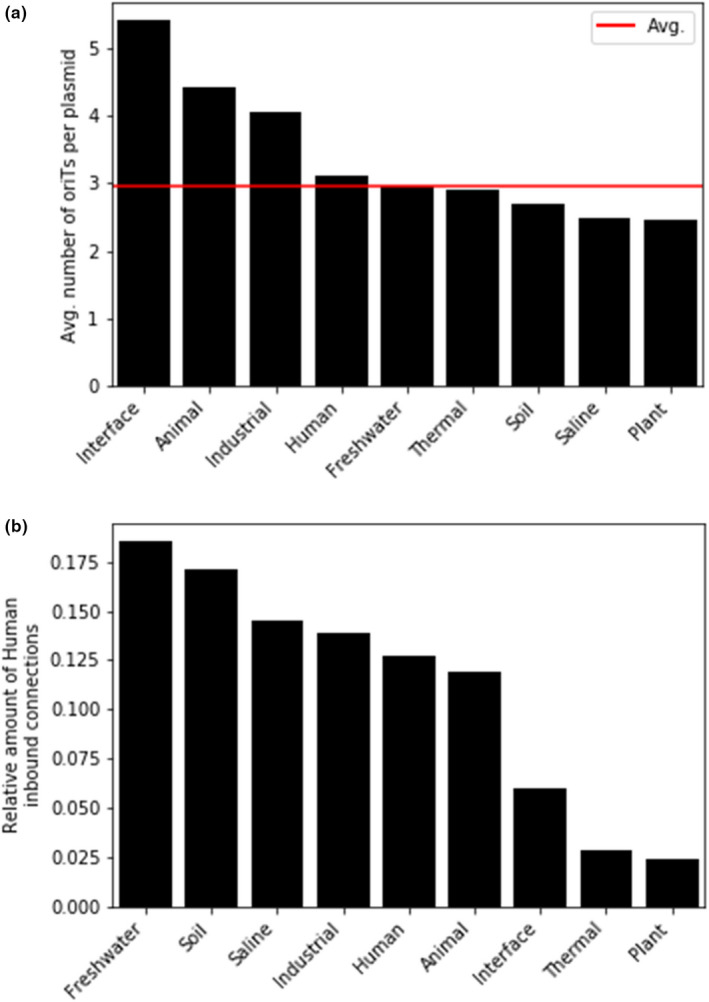
(a) The average number of *oriT*s per plasmid across habitats. (b) The relative amount of connections to human habitat from other environments.

**FIGURE A17:**
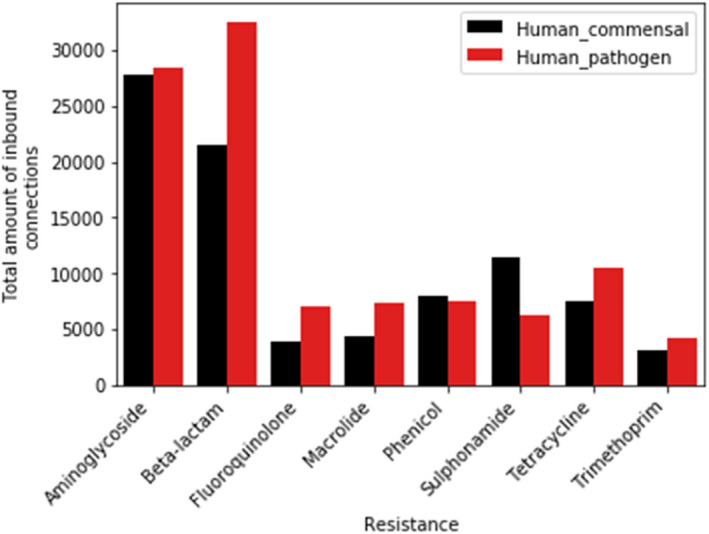
Amount of inbound connections of different AMR classes to the human microbiome. Only AMR classes with more than 2000 connections are shown.

## Data Availability

All data are provided in full in this paper, except for datasets S1, S2, and S3, the software and code that are available in GitHub: https://github.com/JanZrimec/oriT-Strast, as well as the accompanying data available in Zenodo: https://doi.org/10.5281/zenodo.3990609.

## APPENDIX A1

**TABLE A1:** Datasets used in the study.

Task	Dataset	Composition	Availability / Reference
Alignment algorithm testing (Results chapter 1)	s‐distance testing	Balanced dataset of 64 *oriT*s from 4 Mob groups (positives), with 64 negative sequence counterparts	Zrimec and Lapanje (2018)
	Query dataset	106 *oriT*s with known *nic* sites from 4 Mob groups	Dataset S1: https://github.com/JanZrimec/oriT-Strast/blob/master/data/Dataset_S1.csv
	Testing dataset 1	51 plasmids with known *oriT* regions and unknown *nic* sites, from 4 Mob groups	Dataset S2: https://github.com/JanZrimec/oriT-Strast/blob/master/data/Dataset_S2.csv
	Testing dataset 2	13 plasmids with 14 known *nic* sites and unknown Mob groups	Li et al. (2018), Table A3
Searching for *oriT*s (Results chapter 2)	Query dataset	112 *oriT*s with known *nic* sites from 6 Mob groups	Dataset S1: https://github.com/JanZrimec/oriT-Strast/blob/master/data/Dataset_S1.csv
	Target dataset	4602 plasmid sequences with known Mob groups	Shintani et al. (2015), results in Dataset S3: https://github.com/JanZrimec/oriT-Strast/blob/master/data/Dataset_S3.csv
Analysis of hypothetical cross‐habitat network (Results chapter 3)	Dataset of microbial species habitats	3050 distinct species from 927 genera across 9 environmental habitats, with the human habitat, further subdivided into commensal and pathogen subtypes	Pignatelli et al. (2009), Human Microbiome Project Consortium (2012), Escapa et al. (2018), Forster et al. (2016), Dewhirst et al. (2010), Lloyd‐Price et al. (2017)

**TABLE A2:** Performance measures of alignment algorithms for locating *oriT*s and Mob typing with testing dataset 1.

Test	Measure	Strast 220 bp	Strast 40 bp	Blast 220 bp	Blast 40 bp
*OriT* region locating	TPR^a^	1.000	0.818	0.629	0.523
	TNR^b^	0.895	0.944	0.813	0.838
	PPV^c^	0.941	0.964	0.880	0.850
	Accuracy	0.961	0.863	0.686	0.637
	F1‐score	0.970	0.885	0.733	0.648
	MCC^d^	0.918	0.732	0.409	0.355
Mob group typing	TPR	0.968	0.686	0.639	0.551
	TNR	0.800	0.750	0.867	0.939
	PPV	0.882	0.857	0.920	0.950
	Accuracy	0.902	0.706	0.706	0.676
	F1‐score	0.923	0.762	0.754	0.697
	MCC^d^	0.795	0.406	0.461	0.470
Mob subgroup typing	TPR	1.000	0.767	0.639	0.551
	TNR	0.773	0.762	0.867	0.939
	PPV	0.853	0.821	0.920	0.950
	Accuracy	0.902	0.765	0.706	0.676
	F1‐score	0.921	0.793	0.754	0.697
	MCC^d^	0.812	0.523	0.461	0.470

^a^ True‐positive rate, Sensitivity, Recall.

^b^ True‐negative rate, Specificity.

^c^ Positive predictive value, Precision..

^d^ Matthews correlation coefficient.

**TABLE A3:** Strast uncovered *oriT* regions in plasmids of testing dataset 2.

Plasmid name	*OriT* location	*nic* OritDB	*nic* location	Mob	*p*‐value	*nic* (between 5th and 6th nucleotide)
pS7b	[415, 870]	CTTG|CA				
pS7a	[415, 870]	CTTG|CA				
pMAB01	[32885, 33188]	CATCCTG|C	32985	P11	−36.6478	TCCTGCCCGC
pSU233	[105, 383]	GTGGGGTGT|GG	138	F12	−52.3715	GGGTGTGGTG
pMAS2027	[2060, 2078]	TATCCTG|C	2066	P3	−13.3076	ATCCTGCATC
pMAS2027	[38362, 38380]	TATCCTG|C	38375	P3	−19.699	CCTGCATCGC
pKL1	[334, 400]	CATCCTG|T				
pBTK445	[2952, 3002]	CATCCTG|A				
pRS01	[5497, 5536]	CTTG|CA				
Tn916	[2441, 2656]	TGG|T				
LS20	[27531, 27890]	GCCGG|CTTTT				
pRJ6	[2061, 2299]	TGCTTG|CCA	2185	P7	−12.9163	TGCTTGCCAA
Tn1549	[22239, 22266]	RYGCTTG|C				
ICEKp1	[66386, 66635]	GGTTG|GTCGCG	66528	C1	−12.1504	GTTGGTCGCG

**TABLE A4:** OriTfinder Li et al. (2018) uncovered *oriT* regions in plasmids of testing dataset 2.

Plasmid name	*OriT* location	*nic* OritDB	*nic* location	*E*‐value	*nic*
pS7b	[415, 870]	CTTG|CA	[699..708]	<0.01	CTTGCAGTA
pS7a	[415, 870]	CTTG|CA	[698..707]	<0.01	CTTGCAGTA
pMAB01	[32885, 33188]	CATCCTG|C	[32981..32990]	<0.01	CCTGCCCGC
pSU233	[105, 383]	GTGGGGTGT|GG	[151..160]	<0.01	AAACTTGTT
pMAS2027	[38362, 38380]	TATCCTG|C			
pMAS2027	[38362, 38380]	TATCCTG|C			
pKL1	[334, 400]	CATCCTG|T	[430..430]	<0.01	CTGATGCGGG
pBTK445	[2952, 3002]	CATCCTG|A	[2950..2950]	<0.01	ATCACCAGCC
pRS01	[5497, 5536]	CTTG|CA	[5523..5532]	<0.01	CTTGCAAAA
Tn916	[2441, 2656]	TGG|T	[2571..2580]	<0.01	TTGGTTACA
LS20	[27531, 27890]	GCCGG|CTTTT			
pRJ6	[2061, 2299]	TGCTTG|CCA	[2178..2178]	<0.01	CAAACCACTA
Tn1549	[22239, 22266]	RYGCTTG|C			
ICEKp1	[66386, 66635]	GGTTG|GTCGCG	[66444..66453]	<0.01	CGACCAACC

**TABLE A5:** Models of physicochemical and conformational DNA properties.

Variable name	Units	Model	Reference
dG	kcal/mol	Thermodynamic	SantaLucia (1998)
dH	kcal/mol	Thermodynamic	SantaLucia (1998)
dS	cal/K*mol	Thermodynamic	SantaLucia (1998)
dGst	kcal/mol	Thermodynamic	Protozanova et al. (2004)
dGbp	kcal/mol	Thermodynamic	Protozanova et al. (2004)
dGkl	kcal/mol	Thermodynamic	Protozanova et al. (2004)
Tm	deg C	Thermodynamic	Gotoh and Tagashira (1981)
BC	kJ/mol	Thermodynamic	Breslauer et al. (1986)
BA	kJ/mol	Thermodynamic	Aida (1988)
BZ	kJ/mol	Thermodynamic	Ho et al. (1986), Hartmann et al. (1989)
BA_k	kcal/mol	Thermodynamic	Kulkarni and Mukherjee (2013)
dGst_2	kcal/mol	Thermodynamic	Perez et al. (2004)
Zp	A	Unknown	Kulkarni and Mukherjee (2013)
Twist	deg	Curvature	Perez et al. (2004)
Tilt	deg	Curvature	Perez et al. (2004)
Roll	deg	Curvature	Perez et al. (2004)
Shift	A	Curvature	Perez et al. (2004)
Slide	A	Curvature	Perez et al. (2004)
Rise	A	Curvature	Perez et al. (2004)
Phi_slide	kJ mol‐1 A‐2	Curvature	Packer et al. (2000)
Phi_shift	kJ mol‐1 A‐2	Curvature	Packer et al. (2000)
Maj_bend	mu	Curvature	Gartenberg and Crothers (1988)
Min_bend	mu	Curvature	Gartenberg and Crothers (1988)
Wdg	deg	Wedge	Bolshoy and McNamara (1991)
Dir	deg	Wedge	Bolshoy and McNamara (1991)
HT_wdg	deg	Curvature	Kabsch et al. (1982)
ProT2	deg	Curvature	Gorin et al. (1995)
C2	A	Clash function	Gorin et al. (1995)
MajS	A	Clash function	Gorin et al. (1995)
MajD	A	Clash function	Gorin et al. (1995)
MinS	A	Clash function	Gorin et al. (1995)
MinD	A	Clash function	Gorin et al. (1995)
z	nm	Inverse	Geggier and Vologodskii (2010)
h	bp/turn	NN	Geggier and Vologodskii (2010)
z2_set1	nm	Inverse	Sivolob and Khrapunov (1995)
z2_set2	nm	Inverse	Sivolob and Khrapunov (1995)
Deform	deg^3 A^3	NN	Olson et al. (1998)
Twist2	deg	Curvature	Olson et al. (1998)
Tilt2	deg	Curvature	Olson et al. (1998)
Roll2	deg	Curvature	Olson et al. (1998)
Shift2	A	Curvature	Olson et al. (1998)
Slide2	A	Curvature	Olson et al. (1998)
Rise2	A	Curvature	Olson et al. (1998)
u2	cleavage freq.	DNAzeI	Brukner et al. (1995a), Brukner et al. (1995b)
Twist3	deg	Curvature	Karas et al. (1996)
Rise3	A	Curvature	Karas et al. (1996)
Bend	deg	Curvature	Karas et al. (1996)
Tip	deg	Curvature	Karas et al. (1996)
Inclination	deg	Curvature	Karas et al. (1996)
MajWidth	A	Curvature	Karas et al. (1996)
MajDepth	A	Curvature	Karas et al. (1996)
MinWidth	A	Curvature	Karas et al. (1996)
MinDepth	A	Curvature	Karas et al. (1996)
u	cleavage freq.	DNAzeI	Brukner et al. (1995a), Brukner et al. (1995b)
var	fraction	Nucleosome	Satchwell et al. (1986)
phase	deg	Nucleosome	Satchwell et al. (1986)
Roll3	deg	Nucleosome	Goodsell and Dickerson (1994)
MGW	A	DNAshapeR	Rohs et al. (2009), Chiu et al. (2016)
ProT	deg	DNAshapeR	Rohs et al. (2009), Chiu et al. (2016)
Roll	deg	DNAshapeR	Rohs et al. (2009), Chiu et al. (2016)
HelT	deg	DNAshapeR	Rohs et al. (2009), Chiu et al. (2016)
HRC	cleavage intensity	ORChID2	Bishop et al. (2011)
TIDD	no. of events	TIDD	Zrimec and Lapanje (2015)
Tm	deg C	Oligoprop	MATLAB function

**TABLE A6:** Alignment algorithm performance measures.

Measure	Shorthand	Equation
True positives	TP	TP
True negatives	TN	TN
False positives	FP	FP
False negatives	FN	FN
Sensitivity (Recall)	TPR	TP/(TP+FN)
Specificity	TNR	TN/(TN+FP)
Precision	PPV	TP/(TP+FP)
Accuracy	Acc	(TP+TN)/(TP+FP+FN+TN)
F1 score	F1S	2*TP/(2*TP+FP+FN)
Matthews corr. coef.	MCC	(TP*TN‐FP*FN)/sqrt((TP+FP)*(TP+FN)*(TN+FP)*(TN+FN))

**TABLE A7:** Pearson correlation coefficients between sequence identities at all region sizes. All *p*‐values were below 1e‐16.

Region_size2	40	60	90	120	160	220
Region_size1						
40	1	0.940277	0.916119	0.907978	0.889912	0.878428
60	0.940277	1	0.955234	0.940295	0.925025	0.911239
90	0.916119	0.955234	1	0.973206	0.959329	0.944463
120	0.907978	0.940295	0.973206	1	0.979166	0.964006
160	0.889912	0.925025	0.959329	0.979166	1	0.978883
220	0.878428	0.911239	0.944463	0.964006	0.978883	1

**TABLE A8:** Enrichment analysis of plasmid genomic location of *oriT* regions.

Product type	Proportion	Fold change	Fisher's test *p*‐value
Other	0.501689	0.955218	6.41E‐07
Hypothetical protein	0.197835	0.543307	<1E‐16
None	0.21256	4.569195	0.00E+00
Transposition	0.0466	1.323258	4.47E‐10
Conjugation	0.025379	1.166235	1.06E‐02
Integration	0.015938	2.225049	<1E‐16

**TABLE A9:** Top 20 sorted *oriT*s according to the number of co‐occurrences (degree) with other *oriT*s.

Plasmid name	Species	Mob	Mob subgroup	Degree	Degree unique	Num. plasmids	Connected Mob
pNL1	*Novosphingobium aromaticivorans*	F	u	18456	56	1512	(P, V, Q, F, T, C)
BNC1 Plasmid 1	*Mesorhizobium sp.*	Q	2	22651	54	1772	(P, V, Q, F, T, C)
pBBR1	*Bordetella bronchiseptica*	V	2	20875	50	1371	(P, V, Q, F, T, C)
pDOJH10S	*Bifidobacterium longum*	Q	u	24632	42	1617	(P, V, Q, F, T, C)
pKJ50	*Bifidobacterium longum*	Q	u	15585	42	921	(P, V, Q, F, T, C)
pTiC58	*Agrobacterium tumefaciens*	Q	2	13879	41	883	(P, V, Q, F, T, C)
ColE1	*Escherichia coli*	P	5	3179	40	250	(P, V, Q, F, T, C)
pMG160	*Rhodobacter blasticus*	Q	u	11863	38	698	(P, V, Q, F, T, C)
CloDF13	*Escherichia coli*	C	1	6429	36	334	(P, V, Q, F, T, C)
pSymB	*Sinorhizobium meliloti*	Q	2	3535	33	192	(P, V, Q, F, C)
pRetCFN42d	*Rhizobium etli*	Q	2	3608	32	175	(P, V, Q, F, C)
pNGR234a	*Sinorhizobium fredii*	Q	2	3161	30	171	(P, V, Q, F, C)
pTF1	*Acidithiobacillus ferrooxidans*	Q	u	2610	30	136	(P, V, Q, F, C)
pWKS1	*Paracoccus pantotrophus*	V	u	1180	29	70	(P, V, Q, F, C)
pIE1130	*uncultured eubacterium*	Q	1	1425	28	88	(P, V, Q, F, C)
pSymA	*Sinorhizobium meliloti*	Q	2	718	25	40	(P, V, Q, F, C)
pRA2	*Pseudomonas alcaligenes*	P	13	650	24	35	(P, V, Q, F, C)
pOM1	*Oenococcus oeni*	V	u	175	24	19	(P, V, Q, F, C)
p42a	*Rhizobium etli*	Q	2	600	23	31	(P, V, Q, F, C)
pDN1	*Dichelobacter nodosus*	Q	u	476	23	23	(P, V, Q, F, C)

**TABLE A10:** The adjacency matrix of MOB connections across plasmids (note: symmetric across diagonal).

Mob	C	F	P	Q	T	V
C	122	742	195	4371	1	889
F	742	970	664	12637	4	2806
P	195	664	140	3118	1	743
Q	4371	12637	3118	35062	17	15081
T	1	4	1	17	0	2
V	889	2806	743	15081	2	1439

**TABLE A11:** Top 20 sorted connections between Mob subgroups.

Mob subgroup 1	Mob subgroup 2	Num. connections
Q2	Qu	15983
Qu	Qu	10223
Q2	V1	10223
	Fu	8972
Fu	Qu	8972
V2	Qu	7309
Q2	Vu	7309
	Q2	7268
P7	Qu	7268
T1	Qu	3534
Q2	V2	3534
C1	Qu	3274
Q2	C1	3274
V2	Q2	3095
P7	Vu	3095
	Fu	2916
Fu	Q2	2916
V2	Fu	2737
Fu	Vu	2737
T1	Vu	1220

**TABLE A12:** Species and genus count across environmental habitats.

Habitat type	Habitat subtype	Species	Genus
Animal	Animal	1453	1453
Freshwater	Freshwater	2814	2814
Human	Human_commensal	1677	917
	Human_pathogen	293	252
Industrial	Industrial	1879	1879
Interface	Interface	936	936
Plant	Plant	307	307
Saline	Saline	2084	2084
Soil	Soil	2707	2707
Thermal	Thermal	439	439
